# The genome sequence of Greek sea-spurrey, *Spergularia bocconei* (Scheele) Graebn. (Caryophyllaceae)

**DOI:** 10.12688/wellcomeopenres.23609.1

**Published:** 2025-01-30

**Authors:** Denise Power, Maarten J. M. Christenhusz, Colin French, Ian Bennallick, Sahr Mian, Ilia J. Leitch, Royal Botanic Gardens Kew Genome Acquisition Lab, Darwin Tree of Life Barcoding collective, Plant Genome Sizing collective, Wellcome Sanger Institute Tree of Life Management, Samples and Laboratory team, Wellcome Sanger Institute Scientific Operations: Sequencing Operations, Wellcome Sanger Institute Tree of Life Core Informatics team, Tree of Life Core Informatics collective, Darwin Tree of Life Consortium

**Affiliations:** 1 Botanical Society of Britain and Ireland, Camborne, Cornwall, England, UK; 2 Royal Botanic Gardens Kew, Richmond, England, UK; 3 Curtin University, Perth, Western Australia, Australia

**Keywords:** Spergularia bocconei, Boccone's sea-spurrey, Greek sea-spurrey, genome sequence, chromosomal, Caryophyllales

## Abstract

We present a genome assembly from a specimen of *Spergularia bocconei* (Greek sea-spurrey; Streptophyta; Magnoliopsida; Caryophyllales; Caryophyllaceae). The genome sequence has a total length of 466.20 megabases. Most of the assembly is scaffolded into 18 chromosomal pseudomolecules suggesting the individual is an allotetraploid (2 *n* = 4 *x* = 36). The mitochondrial and plastid genome assemblies have lengths of 327.07 kilobases and 152.41 kilobases, respectively.

## Species taxonomy

Eukaryota; Viridiplantae; Streptophyta; Streptophytina; Embryophyta; Tracheophyta; Euphyllophyta; Spermatophyta; Magnoliopsida; Mesangiospermae; eudicotyledons; Gunneridae; Pentapetalae; Caryophyllales; Caryophyllaceae; Sperguleae; *Spergularia*; *Spergularia bocconei* (Scheele) Graebn. (NCBI:txid1053398).

## Background

Greek sea-spurrey, *Spergularia bocconei* (Scheele) Graebn. (Caryophyllaceae), is a small, prostrate, annual/biennial herb found across the Mediterranean Region, Macaronesia, Western Asia and Arabia. It is naturalised in the Azores, Western USA, Baja California, southern South America, South Africa, Tasmania and Korea ( POWO, 2024). The plant often grows in disturbed sandy (sometimes slightly brackish) soils by the sea and in trampled habitats. Indeed, this is the case for the specimen sampled for the Darwin Tree of Life project as it was collected from the compact gravel of the Kynance Cove National Trust parking area on the Lizard Peninsula in Cornwall, UK.

*Spergularia bocconei*’s first appearance in Britain is uncertain although it was first officially recorded in Marazion Green, Cornwall in 1870, and Cornwall still remains the stronghold for this species. It probably arrived with dumped ballast, as it was common to dump ballast in and around the harbours and such dumped waste was a known hotspot for newly arrived alien plants ( Hulme, 2021).

Although the Cornish populations are typically small and vulnerable, they are persistent, and the species is now quite widespread along the coasts, particularly in harbour areas and car parks. Nevertheless, it is also spreading elsewhere in coastal Britain and was reported for the first time in Ireland in 2014, and in Scotland in 2016. These new sites are all associated with either yachting marinas, small harbours or nearby car parks, which strongly suggests it is most frequently being dispersed by boats, cars and pedestrians.

*Spergularia bocconei* is easily confused with the similar, but more common *Spergularia rubra*, which differs in flower colour, stipule shape and size, habit and in being less glandular ( Ratter, 1986; Rich, 2013). Indeed, plants of *S. bocconei* are often covered with soil trapped by their numerous glandular hairs. Various common names have been attributed to this species including Boccone's sea-spurrey named after the Sicilian botanist Paolo Boccone (1633–1704), who was a well-travelled natural historian and described many rare plants.

*Spergularia bocconei* has been reported to have a chromosome count of 2 *n* = 36 ( Kamari *et al.*, 1996), in common with some (e.g. *S. marina*) but not all *Spergularia* species (e.g. *S. media* has 2 *n* = 18) that have been studied. Its chromosome number suggests *S. bocconei* is tetraploid, although it is unclear when it arose. A recent phylogenetic study of Caryophyllaceae (including several *Spergularia* species) suggested that the evolutionary branch leading to *S. bocconei* underwent a whole genome duplication (WGD; i.e., polyploidisation) event somewhere between 8.8 and 2.3 million years ago, whereas there was no evidence of any WGD event on the evolutionary branch leading to extant species reported to have half the number of chromosomes (i.e., 2 *n* = 18) ( Feng *et al.*, 2024).

The genome of *Spergularia bocconei* was sequenced as part of the Darwin Tree of Life Project, a collaborative effort to sequence all named eukaryotic species in the Atlantic Archipelago of Britain and Ireland. Here we present a chromosomal-level genome sequence for *S. bocconei.* Its assembly into 18 pseudochromosomes suggest it is an allopolyploid.

## Genome sequence report

**Figure 1.**
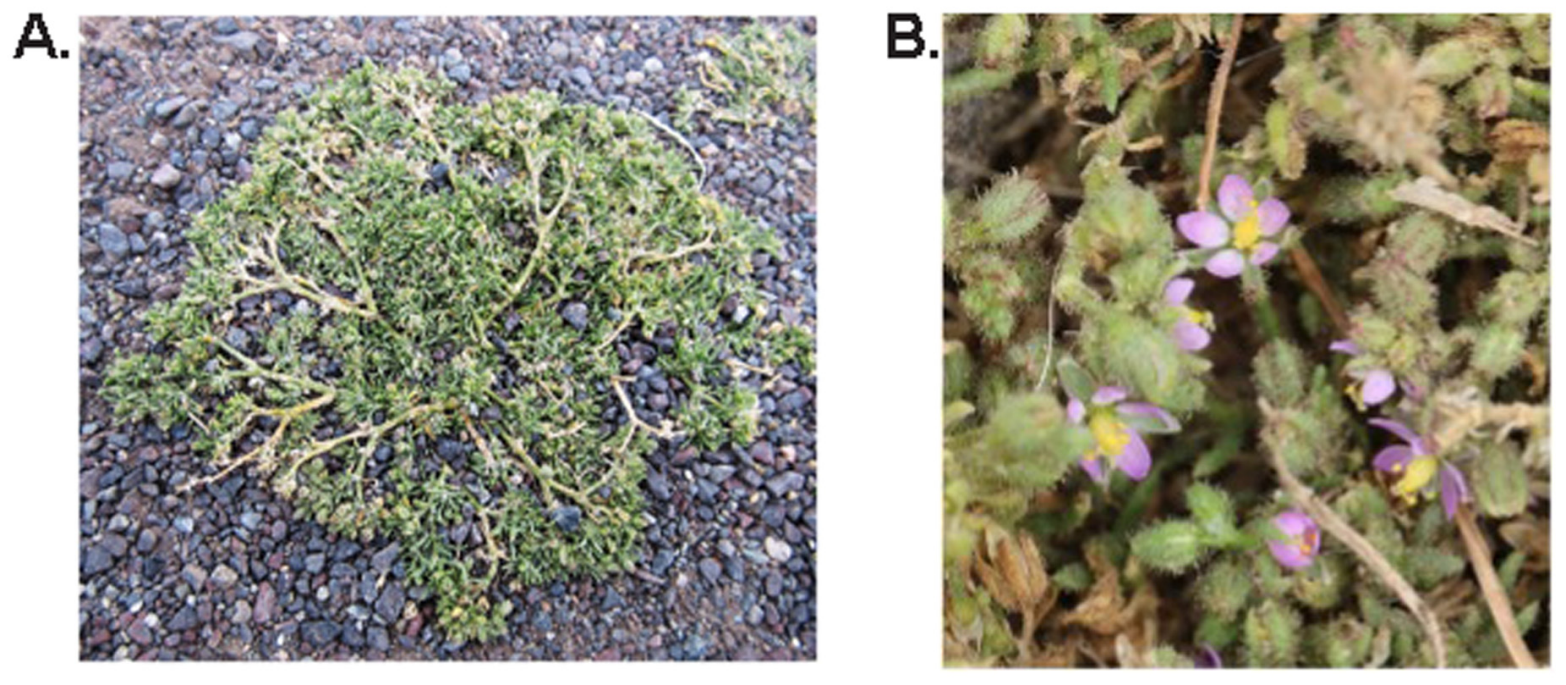
Photograph of ( **a**) the *Spergularia bocconei* (dcSpeBocc1) specimen used for genome sequencing (photo by Peter Wood), and ( **B**) a close up of a different individual showing the flowers and glandular hairs (photo by Colin French).

Using flow cytometry, the genome size (1C-value) of the of *Spergularia bocconei* specimen ( Figure 1) was estimated to be 0.58 pg, equivalent to 560 Mb. The genome was sequenced using Pacific Biosciences single-molecule HiFi long reads, generating a total of 24.38 Gb (gigabases) from 1.97 million reads, providing approximately 86-fold coverage. Primary assembly contigs were scaffolded with chromosome conformation Hi-C data, which produced 131.27 Gb from 869.31 million reads, yielding an approximate coverage of 282-fold. Specimen and sequencing information is summarised in Table 1.

**Table 1.** Specimen and sequencing data for *Spergularia bocconei*.

Project information			
**Study title**	*Spergularia bocconei*		
**Umbrella BioProject**	PRJEB65700		
**Species**	*Spergularia bocconei*		
**BioSample**	SAMEA110450233		
**NCBI taxonomy ID**	1053398		
Specimen information			
**Technology**	**ToLID**	**BioSample accession**	**Organism part**
**PacBio long read sequencing**	dcSpeBocc1	SAMEA110451311	Whole organism
**Hi-C sequencing**	dcSpeBocc1	SAMEA110451311	Whole organism
**RNA sequencing**	dcSpeBocc1	SAMEA110451311	Whole organism
Sequencing information			
**Platform**	**Run accession**	**Read count**	**Base count (Gb)**
**Hi-C Illumina NovaSeq 6000**	ERR12035284	8.69e+08	131.27
**PacBio Sequel IIe**	ERR12015764	1.97e+06	24.38
**RNA Illumina NovaSeq 6000**	ERR12245595	6.42e+07	9.7

**Figure 2.**
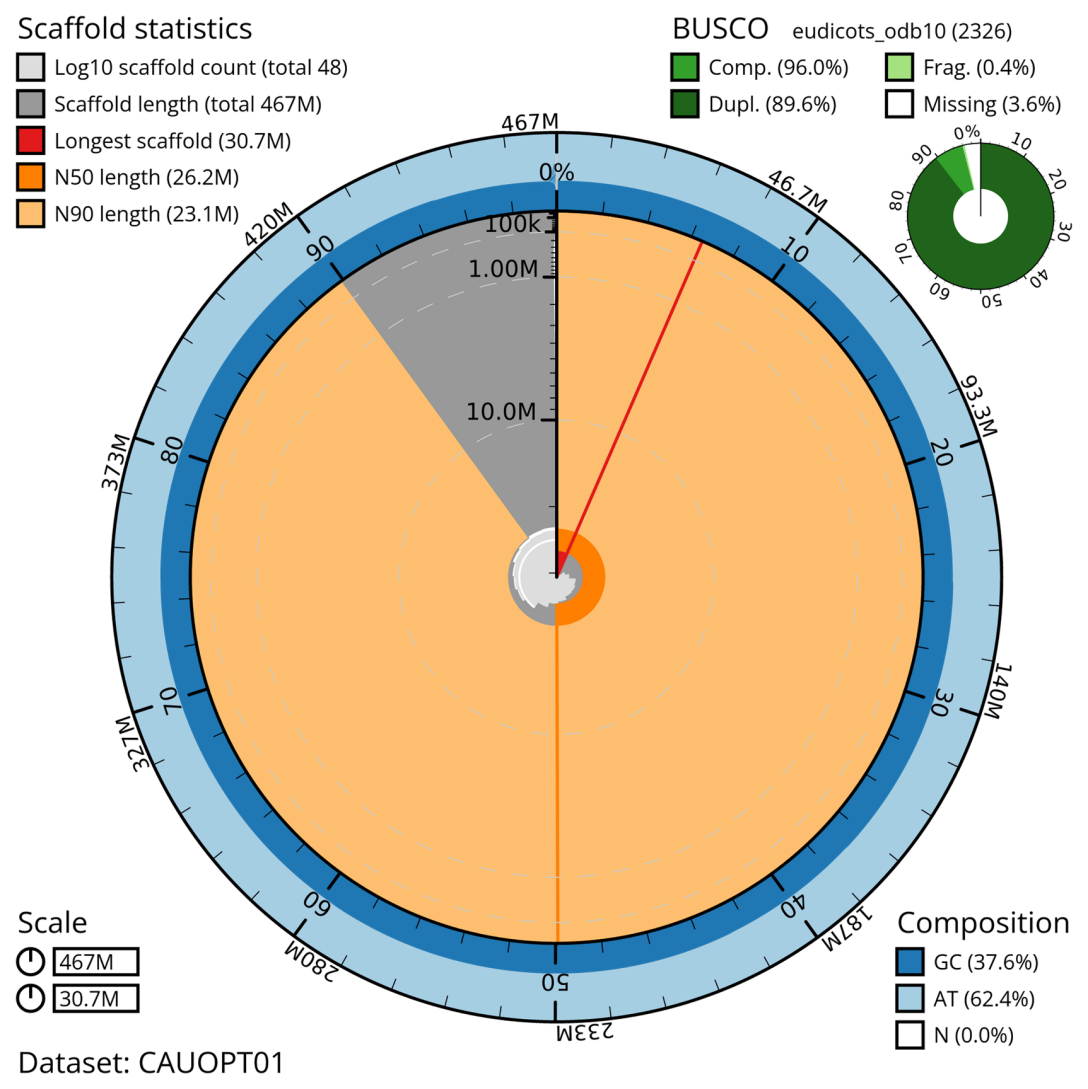
Snail plot summary of assembly statistics for assembly dcSpeBocc1.1: The BlobToolKit snail plot shows N50 metrics and BUSCO gene completeness. The main plot is divided into 1,000 size-ordered bins around the circumference with each bin representing 0.1% of the 466,632,261 bp assembly. The distribution of scaffold lengths is shown in dark grey with the plot radius scaled to the longest scaffold present in the assembly (30,692,203 bp, shown in red). Orange and pale-orange arcs show the N50 and N90 scaffold lengths (26,218,481 and 23,087,913 bp), respectively. The pale grey spiral shows the cumulative scaffold count on a log scale with white scale lines showing successive orders of magnitude. The blue and pale-blue area around the outside of the plot shows the distribution of GC, AT and N percentages in the same bins as the inner plot. A summary of complete, fragmented, duplicated and missing BUSCO genes in the eudicots_odb10 set is shown in the top right. An interactive version of this figure is available at https://blobtoolkit.genomehubs.org/view/CAUOPT01/dataset/CAUOPT01/snail.

**Figure 3.**
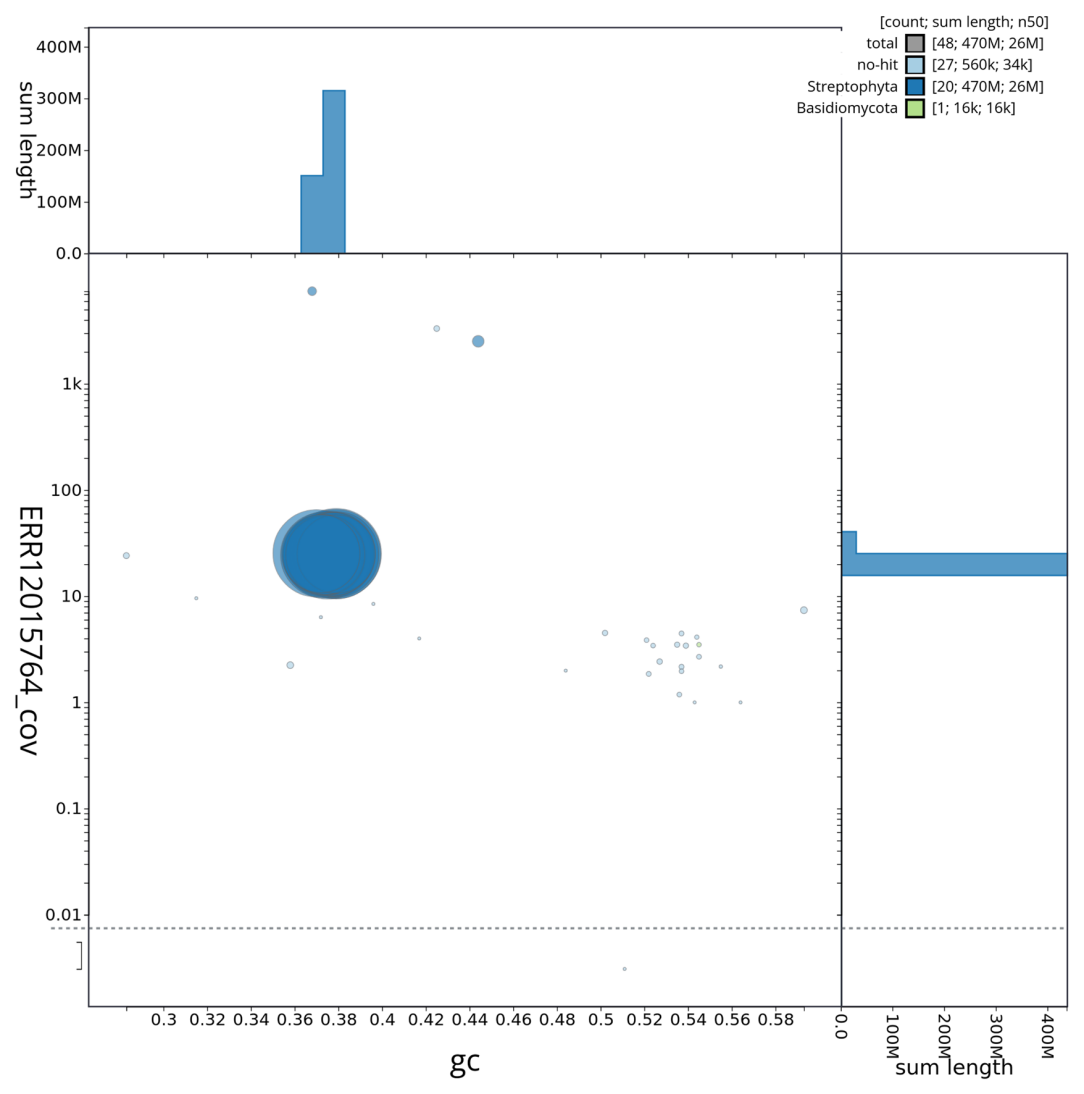
Blob plot of base coverage against GC proportion for sequences in the assembly dcSpeBocc1.1: Scaffolds are coloured by phylum. Circles are sized in proportion to scaffold length. Histograms show the distribution of scaffold length sum along each axis. An interactive version of this figure is available at https://blobtoolkit.genomehubs.org/view/CAUOPT01/dataset/CAUOPT01/blob.

**Figure 4.**
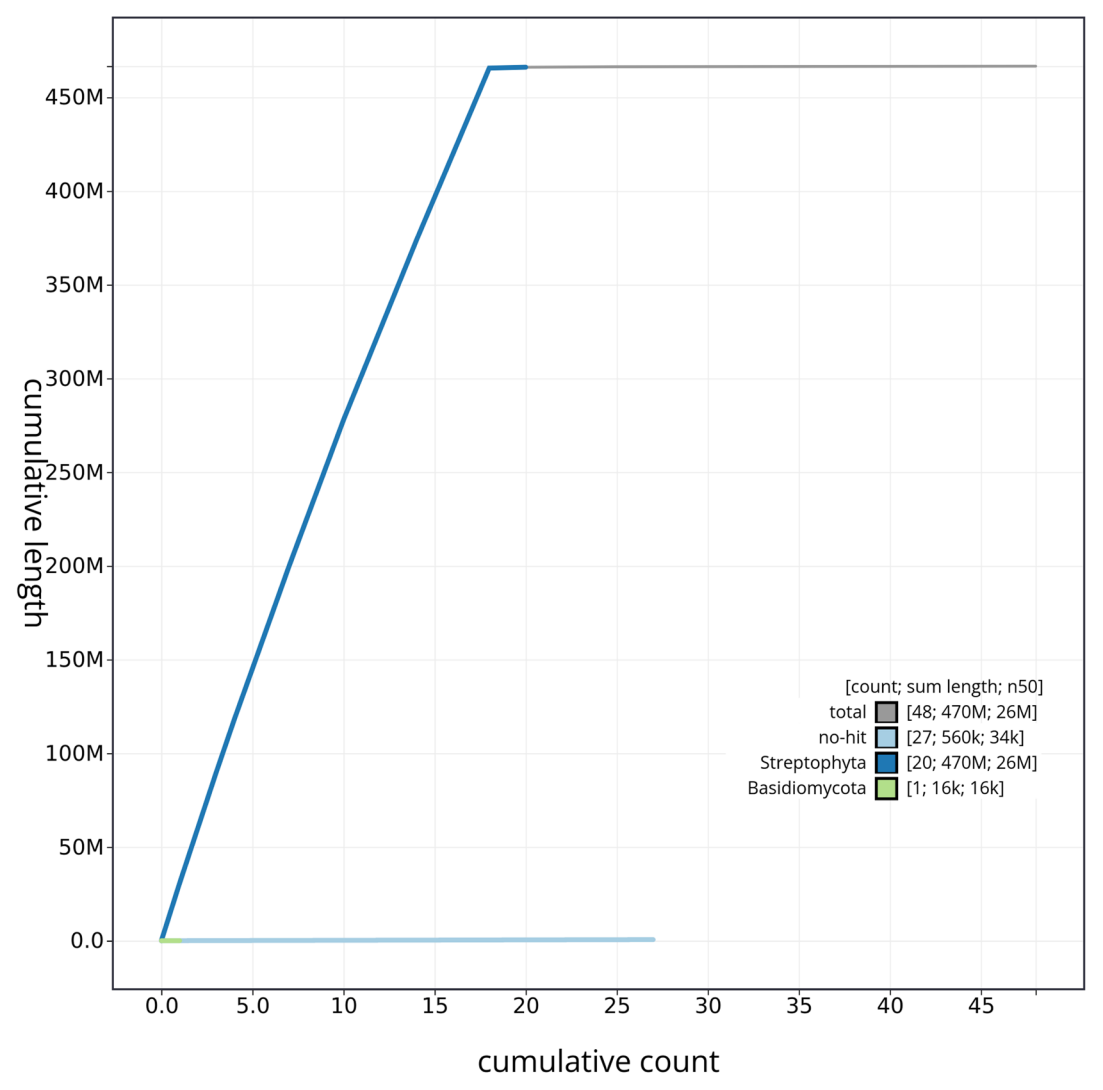
BlobToolKit cumulative sequence plot. The grey line shows cumulative length for all scaffolds. Coloured lines show cumulative lengths of scaffolds assigned to each phylum using the buscogenes taxrule. An interactive version of this figure is available at https://blobtoolkit.genomehubs.org/view/CAUOPT01/dataset/CAUOPT01/cumulative.

**Figure 5.**
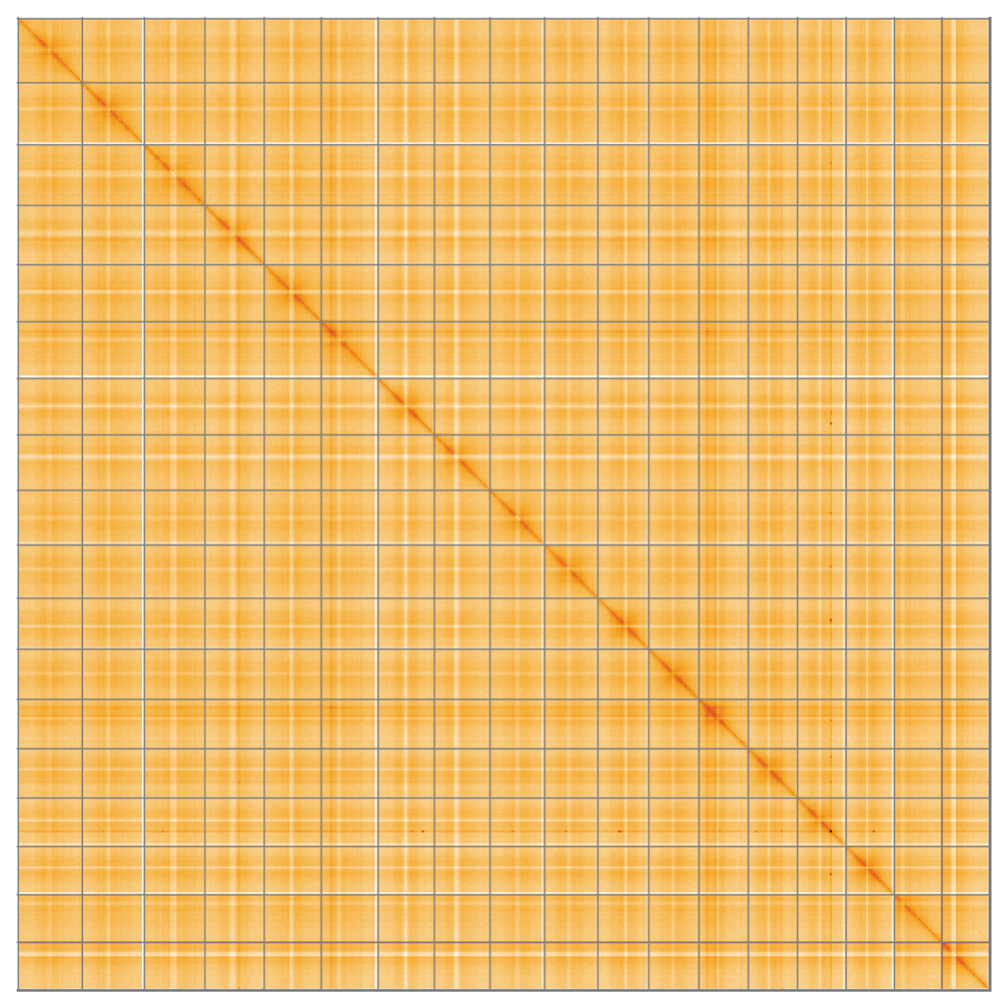
Genome assembly of *Spergularia bocconei*, dcSpeBocc1.1: Hi-C contact map of the dcSpeBocc1.1 assembly, visualised using HiGlass. Chromosomes are shown in order of size from left to right and top to bottom. An interactive version of this figure may be viewed at https://genome-note-higlass.tol.sanger.ac.uk/l/?d=QraDAWETRdypP27HsFlUdw.

Manual assembly curation corrected 30 missing joins or mis-joins, reducing the scaffold number by 4.0%, and decreasing the scaffold N50 by 3.09%. The final assembly has a total length of 466.20 Mb in 46 sequence scaffolds, with 196 gaps. The scaffold N50 is 26.2 Mb ( Table 2) The snail plot in Figure 2 provides a summary of the assembly statistics, while the distribution of assembly scaffolds by GC proportion and coverage is shown in Figure 3. The cumulative assembly plot in Figure 4 shows curves for subsets of scaffolds assigned to different phyla. Most (99.77%) of the assembly sequence was assigned to 18 unique chromosomal-level scaffolds. Chromosome-scale scaffolds confirmed by the Hi-C data are named in order of size ( Figure 5; Table 3). The assembly deposited is of one haplotype. Contigs corresponding to the second haplotype have also been deposited. The mitochondrial and plastid genomes were also assembled and can be found as contigs within the multifasta file of the genome submission.

**Table 2.** Genome assembly data for *Spergularia bocconei*.

Genome assembly		
Assembly name	dcSpeBocc1.1	
Assembly accession	GCA_963457585.1	
*Accession of alternate haplotype*	*GCA_963457555.1*	
Span (Mb)	466.20	
Number of contigs	244	
Number of scaffolds	46	
Longest scaffold (Mb)	30.69	
Assembly metrics *		*Benchmark*
Contig N50 length (Mb)	3.6	*≥ 1 Mb*
Scaffold N50 length (Mb)	26.2	*= chromosome N50*
Consensus quality (QV)	71.3	*≥ 50*
*k*-mer completeness	Primary: 97.94%; alternate: 0.29%; combined: 97.98%	*≥ 95%*
BUSCO **	C:96.0%[S:6.1%,D:89.9%], F:0.4%,M:3.6%,n:2,326	*S > 90%; D > 5%*
Percentage of assembly mapped to chromosomes	99.77%	*≥ 90%*
Organelles	Mitochondrial genome: 327.07 kb; Plastid genome: 152.41 kb	*complete single alleles*

* Assembly metric benchmarks are adapted from Rhie *et al.* (2021) and the Earth BioGenome Project Report on Assembly Standards September 2024. ** BUSCO scores based on the eudicotyledons_odb10 BUSCO set using version 5.4.3. C = complete [S = single copy, D = duplicated], F = fragmented, M = missing, n = number of orthologues in comparison. A full set of BUSCO scores is available at https://blobtoolkit.genomehubs.org/view/CAUOPT01/dataset/CAUOPT01/busco.

**Table 3.** Chromosomal pseudomolecules in the genome assembly of *Spergularia bocconei*, dcSpeBocc1.

INSDC accession	Name	Length (Mb)	GC%
OY734942.1	1	30.69	38.0
OY734943.1	2	29.8	38.0
OY734944.1	3	29.04	37.5
OY734945.1	4	28.46	37.0
OY734946.1	5	27.39	37.5
OY734947.1	6	27.17	38.0
OY734948.1	7	27.06	37.5
OY734949.1	8	26.58	37.5
OY734950.1	9	26.22	37.5
OY734951.1	10	25.49	38.0
OY734952.1	11	24.4	37.5
OY734953.1	12	24.03	37.5
OY734954.1	13	23.77	37.5
OY734955.1	14	23.59	37.5
OY734956.1	15	23.26	37.5
OY734957.1	16	23.09	37.5
OY734958.1	17	22.82	38.0
OY734959.1	18	22.74	37.0
OY734960.1	MT	0.33	44.5
OY734961.1	Pltd	0.15	36.5

The estimated Quality Value (QV) of the final assembly is 71.3. The primary assembly has an estimated *k*-mer completeness of 97.94%, with the alternate haplotype having a completeness of 0.29%, and the combined assemblies have a *k*-mer completeness of 97.98%. The primary assembly also has a BUSCO v5.4.3 completeness of 96.0% (single = 6.1%, duplicated = 89.9%), using the eudicotyledons_odb10 reference set ( *n* = 2,326).

## Methods

### Sample acquisition, DNA barcoding and genome size estimation

A specimen of *Spergularia bocconei* (specimen ID KDTOL10490, ToLID dcSpeBocc1) was collected from Kynance Cove, Cornwall, United Kingdom (latitude 49.97, longitude –5.23) on 2022-06-14. The specimen was collected by Maarten Christenhusz and Denise Power, identified by Maarten Christenhusz and then preserved by freezing at –80 °C. The herbarium voucher of the sequenced plant (K001527018) is deposited in the herbarium of RBG Kew (K).

The initial species identification was verified by an additional DNA barcoding process following the framework developed by Twyford *et al*. (2024). Part of the plant specimen was preserved in silica gel desiccant ( Chase & Hills, 1991). DNA was extracted from the dried specimen, then PCR was used to amplify standard barcode regions. The resulting amplicons were sequenced and compared to public sequence databases including GenBank and the Barcode of Life Database (BOLD). The barcode sequences for this specimen are available on BOLD ( Ratnasingham & Hebert, 2007). Following whole genome sequence generation, DNA barcodes were also used alongside the initial barcoding data for sample tracking through the genome production pipeline at the Wellcome Sanger Institute ( Twyford *et al.*, 2024). The standard operating procedures for the Darwin Tree of Life barcoding have been deposited on protocols.io ( Beasley *et al.*, 2023).

The genome size was estimated by flow cytometry using the fluorochrome propidium iodide and following the ‘one-step’ method as outlined in Pellicer *et al.* (2021). For this species, the General Purpose Buffer (GPB) supplemented with 3% PVP and 0.08% (v/v) beta-mercaptoethanol was used for isolation of nuclei ( Loureiro *et al.*, 2007), and the internal calibration standard was *Solanum lycopersicum* ‘Stupiké polní rané’ with an assumed 1C-value of 968 Mb ( Doležel *et al.*, 2007).

### Nucleic acid extraction

The workflow for high molecular weight (HMW) DNA extraction at the WSI Tree of Life Core Laboratory includes a sequence of core procedures: sample preparation and homogenisation, DNA extraction, fragmentation and purification. Detailed protocols are available on protocols.io ( Denton *et al.*, 2023). The dcSpeBocc1 sample was weighed and dissected on dry ice ( Jay *et al.*, 2023), and tissue from the whole organism was cryogenically disrupted using the Covaris cryoPREP ^®^ Automated Dry Pulverizer ( Narváez-Gómez *et al.*, 2023).

HMW DNA was extracted in the WSI Scientific Operations core using the Automated Plant MagAttract v2 protocol ( Todorovic *et al.*, 2023). HMW DNA was sheared into an average fragment size of 12–20 kb in a Megaruptor 3 system ( Bates *et al.*, 2023). Sheared DNA was purified by solid-phase reversible immobilisation, using AMPure PB beads to eliminate shorter fragments and concentrate the DNA ( Strickland *et al.*, 2023). The concentration of the sheared and purified DNA was assessed using a Nanodrop spectrophotometer and Qubit Fluorometer and Qubit dsDNA High Sensitivity Assay kit. Fragment size distribution was evaluated by running the sample on the FemtoPulse system.

RNA was extracted from the dcSpeBocc1 sample in the Tree of Life Laboratory at the WSI using the RNA Extraction: Automated MagMax™ *mir*Vana protocol ( do Amaral *et al.*, 2023). The RNA concentration was assessed using a Nanodrop spectrophotometer and a Qubit Fluorometer using the Qubit RNA Broad-Range Assay kit. Analysis of the integrity of the RNA was done using the Agilent RNA 6000 Pico Kit and Eukaryotic Total RNA assay.

### Hi-C tissue preparation

Hi-C data were generated from tissue from the dcSpeBocc1 sample using the Arima-HiC v2 kit. Tissue was finely ground using cryoPREP and then subjected to nuclei isolation. Nuclei were isolated using a modified protocol of the Qiagen QProteome Cell Compartment Kit where only CE1 and CE2 buffers are used in combination with QiaShredder spin columns. After isolation, the nuclei were fixed using 37% formaldehyde solution to crosslink the DNA. The crosslinked DNA was then digested using the restriction enzyme master mix. The 5’-overhangs were then filled in and labelled with biotinylated nucleotides and proximally ligated. An overnight incubation was carried out for enzymes to digest remaining proteins and for crosslinks to reverse. A clean up was performed with SPRIselect beads prior to library preparation. DNA concentration was quantified using the Qubit Fluorometer v4.0 and Qubit HS Assay Kit according to the manufacturer’s instructions.

### Library preparation and sequencing

Pacific Biosciences HiFi circular consensus DNA sequencing libraries were constructed according to the manufacturers’ instructions. Poly(A) RNA-Seq libraries were constructed using the NEB Ultra II RNA Library Prep kit. DNA and RNA sequencing was performed by the Scientific Operations core at the WSI on Pacific Biosciences Sequel IIe (HiFi) and Illumina NovaSeq 6000 (RNA-Seq) instruments.

For Hi-C library preparation, DNA was fragmented to a size of 400 to 600 bp using a Covaris E220 sonicator. The DNA was then enriched, barcoded, and amplified using the NEBNext Ultra II DNA Library Prep Kit (New England Biolabs) following manufacturer’s instructions. Hi-C sequencing was performed using paired-end sequencing with a read length of 150 bp on an Illumina NovaSeq 6000 instrument.

### Genome assembly, curation and evaluation

***Assembly***

The HiFi reads were first assembled using Hifiasm ( Cheng *et al.*, 2021) with the --primary option. Haplotypic duplications were identified and removed using purge_dups ( Guan *et al.*, 2020). The Hi-C reads were mapped to the primary contigs using bwa-mem2 ( Vasimuddin *et al.*, 2019). The contigs were further scaffolded using the provided Hi-C data ( Rao *et al.*, 2014) in YaHS ( Zhou *et al.*, 2023) using the --break option. The scaffolded assemblies were evaluated using Gfastats ( Formenti *et al.*, 2022), BUSCO ( Manni *et al.*, 2021) and MERQURY.FK ( Rhie *et al.*, 2020). The organelle genomes were assembled using OATK ( Zhou, 2023).

***Curation***

The assembly was decontaminated using the Assembly Screen for Cobionts and Contaminants (ASCC) pipeline (article in preparation). Flat files and maps used in curation were generated in TreeVal ( Pointon *et al.*, 2023). Manual curation was primarily conducted using PretextView ( Harry, 2022), with additional insights provided by JBrowse2 ( Diesh *et al.*, 2023) and HiGlass ( Kerpedjiev *et al.*, 2018). Scaffolds were visually inspected and corrected as described by Howe *et al*. (2021). Any identified contamination, missed joins, and mis-joins were corrected, and duplicate sequences were tagged and removed. The process is documented at https://gitlab.com/wtsi-grit/rapid-curation (article in preparation).

***Evaluation of final assembly***

The Merqury.FK tool ( Rhie *et al.*, 2020) was used to evaluate *k*-mer completeness and assembly quality for the primary and alternate haplotypes using the *k*-mer databases ( *k* = 31) that were pre-computed prior to genome assembly. The analysis outputs included assembly QV scores and completeness statistics.

A Hi-C contact map was produced for the final, public version of the assembly. The Hi-C reads were aligned using bwa-mem2 ( Vasimuddin *et al.*, 2019) and the alignment files were combined using SAMtools ( Danecek *et al.*, 2021). The Hi-C alignments were converted into a contact map using BEDTools ( Quinlan & Hall, 2010) and the Cooler tool suite ( Abdennur & Mirny, 2020). The contact map is visualised in HiGlass ( Kerpedjiev *et al.*, 2018).

The blobtoolkit pipeline is a Nextflow port of the previous Snakemake Blobtoolkit pipeline ( Challis *et al.*, 2020). It aligns the PacBio reads in SAMtools and minimap2 ( Li, 2018) and generates coverage tracks for regions of fixed size. In parallel, it queries the GoaT database ( Challis *et al.*, 2023) to identify all matching BUSCO lineages to run BUSCO ( Manni *et al.*, 2021). For the three domain-level BUSCO lineages, the pipeline aligns the BUSCO genes to the UniProt Reference Proteomes database ( Bateman *et al.*, 2023) with DIAMOND blastp ( Buchfink *et al.*, 2021). The genome is also divided into chunks according to the density of the BUSCO genes from the closest taxonomic lineage, and each chunk is aligned to the UniProt Reference Proteomes database using DIAMOND blastx. Genome sequences without a hit are chunked using seqtk and aligned to the NT database with blastn ( Altschul *et al.*, 1990). The blobtools suite combines all these outputs into a blobdir for visualisation.

The genome assembly and evaluation pipelines were developed using nf-core tooling ( Ewels *et al.*, 2020) and MultiQC ( Ewels *et al.*, 2016), relying on the Conda package manager, the Bioconda initiative ( Grüning *et al.*, 2018), the Biocontainers infrastructure ( da Veiga Leprevost *et al.*, 2017), as well as the Docker ( Merkel, 2014) and Singularity ( Kurtzer *et al.*, 2017) containerisation solutions.

Table 4 contains a list of relevant software tool versions and sources.

**Table 4.** Software tools: versions and sources.

Software tool	Version	Source
BEDTools	2.30.0	https://github.com/arq5x/bedtools2
Blast	2.14.0	ftp://ftp.ncbi.nlm.nih.gov/blast/executables/blast+/
BlobToolKit	4.3.7	https://github.com/blobtoolkit/blobtoolkit
BUSCO	5.4.3 and 5.5.0	https://gitlab.com/ezlab/busco
bwa-mem2	2.2.1	https://github.com/bwa-mem2/bwa-mem2
Cooler	0.8.11	https://github.com/open2c/cooler
DIAMOND	2.1.8	https://github.com/bbuchfink/diamond
fasta_ windows	0.2.4	https://github.com/tolkit/fasta_windows
FastK	427104ea91c78c3b8b8b49f1a7d6bbeaa869ba1c	https://github.com/thegenemyers/FASTK
Gfastats	1.3.6	https://github.com/vgl-hub/gfastats
GoaT CLI	0.2.5	https://github.com/genomehubs/goat-cli
Hifiasm	0.16.1-r375	https://github.com/chhylp123/hifiasm
HiGlass	44086069ee7d4d3f6f3f0012569789ec138f42b84 aa44357826c0b6753eb28de	https://github.com/higlass/higlass
Merqury.FK	d00d98157618f4e8d1a9190026b19b471055b2 2e	https://github.com/thegenemyers/MERQURY.FK
MultiQC	1.14, 1.17, and 1.18	https://github.com/MultiQC/MultiQC
NCBI Datasets	15.12.0	https://github.com/ncbi/datasets
Nextflow	23.04.0-5857	https://github.com/nextflow-io/nextflow
PretextView	0.2	https://github.com/sanger-tol/PretextView
OATK	0.2	https://github.com/c-zhou/oatk
purge_dups	1.2.3	https://github.com/dfguan/purge_dups
samtools	1.16.1, 1.17, and 1.18	https://github.com/samtools/samtools
sanger-tol/ ascc	-	https://github.com/sanger-tol/ascc
Seqtk	1.3	https://github.com/lh3/seqtk
Singularity	3.9.0	https://github.com/sylabs/singularity
TreeVal	1.0.0	https://github.com/sanger-tol/treeval
YaHS	1.1a.2	https://github.com/c-zhou/yahs

### Wellcome Sanger Institute – Legal and Governance

The materials that have contributed to this genome note have been supplied by a Darwin Tree of Life Partner. The submission of materials by a Darwin Tree of Life Partner is subject to the **‘Darwin Tree of Life Project Sampling Code of Practice’**, which can be found in full on the Darwin Tree of Life website here. By agreeing with and signing up to the Sampling Code of Practice, the Darwin Tree of Life Partner agrees they will meet the legal and ethical requirements and standards set out within this document in respect of all samples acquired for, and supplied to, the Darwin Tree of Life Project.

Further, the Wellcome Sanger Institute employs a process whereby due diligence is carried out proportionate to the nature of the materials themselves, and the circumstances under which they have been/are to be collected and provided for use. The purpose of this is to address and mitigate any potential legal and/or ethical implications of receipt and use of the materials as part of the research project, and to ensure that in doing so we align with best practice wherever possible. The overarching areas of consideration are:

•   Ethical review of provenance and sourcing of the material

•   Legality of collection, transfer and use (national and international)

Each transfer of samples is further undertaken according to a Research Collaboration Agreement or Material Transfer Agreement entered into by the Darwin Tree of Life Partner, Genome Research Limited (operating as the Wellcome Sanger Institute), and in some circumstances other Darwin Tree of Life collaborators.

## Data Availability

European Nucleotide Archive: *Spergularia bocconei*. Accession number PRJEB65700; https://identifiers.org/ena.embl/PRJEB65700. The genome sequence is released openly for reuse. The *Spergularia bocconei* genome sequencing initiative is part of the Darwin Tree of Life (DToL) project. All raw sequence data and the assembly have been deposited in INSDC databases. The genome will be annotated using available RNA-Seq data and presented through the Ensembl pipeline at the European Bioinformatics Institute. Raw data and assembly accession identifiers are reported in Table 1.

## Author information

Members of the Royal Botanic Gardens Kew Genome Acquisition Lab are listed here: https://doi.org/10.5281/zenodo.12625079.

Members of the Plant Genome Sizing collective are listed here: https://doi.org/10.5281/zenodo.7994306.

Members of the Darwin Tree of Life Barcoding collective are listed here: https://doi.org/10.5281/zenodo.12158331

Members of the Wellcome Sanger Institute Tree of Life Management, Samples and Laboratory team are listed here: https://doi.org/10.5281/zenodo.12162482.

Members of Wellcome Sanger Institute Scientific Operations: Sequencing Operations are listed here: https://doi.org/10.5281/zenodo.12165051.

Members of the Wellcome Sanger Institute Tree of Life Core Informatics team are listed here: https://doi.org/10.5281/zenodo.12160324.

Members of the Tree of Life Core Informatics collective are listed here: https://doi.org/10.5281/zenodo.12205391.

Members of the Darwin Tree of Life Consortium are listed here: https://doi.org/10.5281/zenodo.4783558.
