# Supplementary material for: The genome sequence of Greek sea-spurrey, Spergularia bocconei (Scheele) Graebn. (Caryophyllaceae)
Source: Wellcome Open Res. 2025 Jan 30;10:34. [Version 1] doi: 10.12688/wellcomeopenres.23609.1 (PMC11904401; doi:10.12688/wellcomeopenres.23609.1)
Supplement: Peer review history

# Reviewer report: Reviewer response for version 1

**DOI:** 10.21956/wellcomeopenres.26045.r118029

**Published:** 2025-03-12

**Authors:** Joseph F. Walker

**Affiliations:** 1 University of Illinois at Chicago, Chicago, Illinois, USA

The article provides an overview of the sequencing and assembly procedure for the Spergularia bocconei genome and is a descriptive overview of the genome, plastome, and mitogenome.

 The authors have ensured an herbarium voucher is created for the sample and the website details where the sample was collected from. As this is part of the larger Darwin Tree of Life project, the authors have followed similar sequencing and assembly pipeline procedure methods as many other previously published genomes.

 The release on the website mentions transcriptomic data, however, this appears to only be raw sequencing data and is not discussed in much detail in the paper. Along with the raw sequencing data, it would be helpful for researchers if an assembled transcriptome was provided. Furthermore, annotations would be helpful. It's mentioned in the Data Availability that this will be done, but without the annotations, the use of it is limited. In short, I would recommend providing a transcriptome and annotations before officially releasing the genome.

 Aside from these small comments, the authors provide some interesting insights into the background of this species and do a good job of presenting the genome.

Are sufficient details of methods and materials provided to allow replication by others?

Yes

Is the rationale for creating the dataset(s) clearly described?

Yes

Are the datasets clearly presented in a useable and accessible format?

Yes

Are the protocols appropriate and is the work technically sound?

Yes

Reviewer Expertise:

phylogenomics

I confirm that I have read this submission and believe that I have an appropriate level of expertise to confirm that it is of an acceptable scientific standard.

# Reviewer report: Reviewer response for version 1

**DOI:** 10.21956/wellcomeopenres.26045.r118275

**Published:** 2025-03-10

**Authors:** Bimal K Chetri

**Affiliations:** 1 Environment and Life Sciences, Sherubtse College, Kanglung, Trashigang, Bhutan

This manuscript details a comprehensive chromosome-level genome assembly of *Spergularia bocconei*, an allotetraploid plant species from the Caryophyllaceae family, sequenced as part of the Darwin Tree of Life Project. The 466.20 Mb genome assembly is organized into 18 chromosomal pseudomolecules, indicating an allotetraploid nature (2n = 4x = 36). The assembly was achieved using PacBio HiFi long-read sequencing, complemented by Hi-C scaffolding and RNA sequencing to enhance genome structure and annotation. Additionally, the mitochondrial and plastid genomes were assembled. While the study is well-executed and provides a valuable genomic resource, it highlights areas needing improvement, particularly in functional annotation, comparative genomics, and data presentation. The authors justify sequencing *Spergularia bocconei* by linking it to the broader Darwin Tree of Life Project, emphasizing the species' taxonomic, ecological, and evolutionary significance, especially its polyploid nature. The study also addresses the challenges of distinguishing *S. bocconei* from closely related species and the importance of understanding its genome structure. Employing best-practice sequencing technologies, including PacBio HiFi reads, Hi-C scaffolding, and Illumina RNA sequencing, the genome assembly process is robust, utilizing Hifiasm for primary assembly, Hi-C data for scaffolding, and BUSCO for quality assessment. The genome assembly achieves high completeness (BUSCO score: 96%), indicating strong data reliability.

Are sufficient details of methods and materials provided to allow replication by others?

Yes

Is the rationale for creating the dataset(s) clearly described?

Yes

Are the datasets clearly presented in a useable and accessible format?

Yes

Are the protocols appropriate and is the work technically sound?

Yes

Reviewer Expertise:

Plant organellar genomics and molecular phylogeny

I confirm that I have read this submission and believe that I have an appropriate level of expertise to confirm that it is of an acceptable scientific standard.

# Reviewer report: Reviewer response for version 1

**DOI:** 10.21956/wellcomeopenres.26045.r118284

**Published:** 2025-02-13

**Authors:** Ahmed Faried

**Affiliations:** 1 Biology, Shaqra University, Shaqra, Riyadh Province, Saudi Arabia; 2 Assiut University, Assiut, Egypt

**1. Summary of the Article**

 This manuscript presents a high-quality, chromosome-level genome assembly of *Spergularia bocconei*, an allotetraploid plant species from the Caryophyllaceae family. The genome was sequenced as part of the Darwin Tree of Life Project, an initiative to sequence all eukaryotic species in Britain and Ireland. The 466.20 Mb genome assembly is scaffolded into 18 chromosomal pseudomolecules, suggesting an allotetraploid nature (2n = 4x = 36). The assembly was generated using PacBio HiFi long-read sequencing, with additional Hi-C scaffolding and RNA sequencing to improve genome structure and annotation. The mitochondrial and plastid genomes were also assembled.

 The study is well-executed and provides a valuable genomic resource, but certain areas require improvement, particularly in functional annotation, comparative genomics, and data presentation. Below, I address the key review criteria and provide constructive feedback for improving the manuscript.

**2. Detailed Evaluation**

**Q1: Is the rationale for creating the dataset(s) clearly described?**

**Yes**

 The authors provide a clear justification for sequencing *Spergularia bocconei*, linking it to the broader Darwin Tree of Life Project.

 The manuscript contextualizes the species' taxonomic, ecological, and evolutionary relevance, emphasizing its polyploid nature and its importance in plant genomics.

 The study also highlights the challenges of distinguishing *S. bocconei* from closely related species and the significance of understanding its genome structure.

**Q2: Are the protocols appropriate and is the work technically sound?**

**Yes**

 The study employs best-practice sequencing technologies:

 PacBio HiFi reads for long-read sequencing.

 Hi-C scaffolding for chromosome-level assembly.

 Illumina RNA sequencing for transcriptome validation.

 The genome assembly process is robust, using Hifiasm for primary assembly, Hi-C data for scaffolding, and BUSCO for quality assessment.

 The genome assembly achieves high completeness (BUSCO score: 96%), indicating strong data reliability.

**Q3: Are sufficient details of methods and materials provided to allow replication by others?**

**Partly**

 While the manuscript provides extensive methodological details, some areas require clarification or expansion to ensure full reproducibility:

**Key Areas Needing Improvement:**

**1- Contamination Filtering and Assembly Curation**

 The manuscript mentions manual curation and decontamination using the ASCC pipeline, but does not describe:

 The criteria used to identify contaminants.

 The extent of contaminant removal.

 Any potential biases introduced by these steps.

 * **Suggested Improvement:**

 Provide details on how contamination was detected and removed.

 If possible, include a summary of contaminant sequences filtered out.

**2- Functional Annotation and Gene Prediction**

 The genome assembly is high quality, but no detailed gene annotation is provided.

* Suggested Improvement:

 Perform functional annotation using InterProScan, Pfam, and KEGG to predict gene functions.

 Identify key stress-adaptation genes, given the species' coastal habitat.

 Discuss genes related to salinity tolerance and polyploidy evolution.

**3- Transcriptomic Validation of Genes**

 The study mentions RNA sequencing, but does not describe:

 How RNA data were used for gene annotation.

 Whether alternative splicing or gene expression levels were analyzed.

*Suggested Improvement:

 Map RNA-Seq reads to the genome to confirm gene structures.

 Perform differential gene expression analysis in different tissues or environmental conditions.

**4-Comparative Genomics Analysis**

 The study does not include a comparative analysis of *S. bocconei* with closely related species.

*Suggested Improvement:

 Compare genome structure with S. marina (2n = 36) and S. media (2n = 18).

 Perform synteny analysis to identify chromosomal rearrangements.

 * These improvements are necessary to make the study fully replicable and scientifically sound.

**Q4: Are the datasets clearly presented in a usable and accessible format?**

**Partly**

 The genome assembly and raw sequence data are publicly available in the European Nucleotide Archive (ENA), but some aspects of data usability could be improved.

**Key Areas Needing Improvement:**

**1- Lack of Functional Annotation Data**

 No gene models or annotation files are provided for download.

*Suggested Improvement:

 Upload gene annotation files to a public repository (e.g., Ensembl, NCBI).

 Provide a browser-based visualization (e.g., UCSC Genome Browser).

**2- Data Accessibility and Usability**

 While raw genome data are accessible, the manuscript does not explain how users can explore the genome.

*Suggested Improvement:

 Add links to interactive genome browsers (e.g., Ensembl Plants).

 Provide a README file describing dataset contents.

 *These improvements are necessary to maximize the usability of the dataset.

**3. Key Revisions Required**

 To make the manuscript scientifically sound, the authors must address the following issues:

 1- Expand the Methods Section to clearly describe contamination filtering and assembly curation steps.

 2- Perform functional annotation of genes using InterProScan, KEGG, and Pfam.

 3- Use RNA sequencing data to validate gene predictions and analyze gene expression.

 4- Include a comparative genomic analysis with related *Spergularia* species.

**4. Final Recommendation**

 This study presents an important and high-quality genome assembly, but several critical issues must be addressed before indexing. The lack of functional annotation and comparative genomics limits the biological insights that can be drawn from the dataset.

**Recommended Decision:**

 Revisions Required (Minor Revision).

Are sufficient details of methods and materials provided to allow replication by others?

Partly

Is the rationale for creating the dataset(s) clearly described?

Yes

Are the datasets clearly presented in a useable and accessible format?

Partly

Are the protocols appropriate and is the work technically sound?

Yes

Reviewer Expertise:

The manuscript requires several specific but significant revisions before full approval:Insufficient Functional AnnotationTranscriptomic Data Not Fully IntegratedComparative Genomics Missing

I confirm that I have read this submission and believe that I have an appropriate level of expertise to confirm that it is of an acceptable scientific standard, however I have significant reservations, as outlined above.
